# Antifungal activity and mode of action of thymol and its synergism with nystatin against *Candida* species involved with infections in the oral cavity: an *in vitro* study

**DOI:** 10.1186/s12906-015-0947-2

**Published:** 2015-11-24

**Authors:** Ricardo Dias de Castro, Trícia Murielly Pereira Andrade de Souza, Louise Morais Dornelas Bezerra, Gabriela Lacet Silva Ferreira, Edja Maria Melo de Brito Costa, Alessandro Leite Cavalcanti

**Affiliations:** Postgraduate Program in Dentistry, School of Dentistry, Universidade Federal da Paraíba, Campus I, ZIP: 58.051-900 Joao Pessoa, Paraiba Brazil; School of Dentistry, Universidade Federal da Paraíba, ZIP: 58.051-900 Joao Pessoa, Paraiba Brazil; Post-Graduate Program in Dentistry, State University of Paraiba, Campina Grande, Paraiba Brazil

**Keywords:** Microbiology, *Candida albicans*, Thymol

## Abstract

**Background:**

Limitations of antifungal agents used in the treatment of oral candidiasis, as the development of resistant strains, are known by the scientific community. In this context, the aim of this study was to evaluate the antifungal activity of thymol against *Candida albicans, Candida tropicalis* and *Candida krusei* strains and to determine its mode of action and synergistic effect when combined with the synthetic antifungal nystatin.

**Methods:**

The minimum inhibitory concentration (MIC) was determined using a microdilution technique, and the minimum fungicidal concentration (MFC) was determined via subculture sowing. The mode of action of thymol was established by verifying fungal growth in the presence of sorbitol or ergosterol. The fractional inhibitory concentration index (FIC) was determined using the checkerboard method.

**Results:**

Thymol presented an antifungal effect, with MICs of 39 μg/mL for *C. albicans* and *C. krusei* and 78 μg/mL for *C. tropicalis*. The results of the antifungal test remained unchanged in the presence of sorbitol; however, the MIC value of thymol against *C. albicans* increased eight times (from 39.0 to 312.5 μg/mL) in presence of exogenous ergosterol. The combination of thymol and nystatin reduced the MIC values of both products by 87.4 %, generating an FIC index of 0.25.

**Conclusions:**

Thymol was found to have a fungicidal effect on *Candida* species and a synergistic effect when combined with nystatin.

## Background

The emergence of opportunistic fungal infections, especially in immunocompromised individuals, highlights the need to elucidate new therapeutic options, especially because the microorganisms involved usually have remarkable morphological plasticity and express genes involved in the mechanisms of resistance to antifungal agents [1].

Oral candidiasis is a superficial infection that affects the palate region and jugal mucosa. It is notable for its high prevalence and the limited therapeutic tools available for treatment. It is an infection produced by microorganisms of the genus *Candida*; *Candida albicans* is the most common and most pathogenic species [2–4], although *Candida tropicalis* and *Candida krusei* have important roles in the development of the disease [5, 6].

Local and systemic factors may predispose individuals or trigger clinical cases of oral candidiasis. Such factors include saliva acidity, hyposalivation, nightly use of dental prostheses, endocrine disorders, nutritional deficiencies, smoking, poor oral hygiene, immunosuppressive drug use and radiotherapy and chemotherapy treatment of maxillo-facial structures [7].

The use of nystatin to treat tissue injury has been recommended [8], although other substances, such as miconazole, fluconazole, and ketoconazole may be prescribed instead [9–11]. However, indiscriminate use and the small number of available antifungal agents have promoted the development of resistant strains, especially in immunocompromised individuals [12]. This fact justifies the development of new therapies for use in clinical practice [13, 14].

Among these new therapies, natural products stand out; they are considered sources of bioactive molecules with potential therapeutic applications in medicine and dentistry [15, 16]. Many studies have been conducted on the antifungal activity of natural products against *Candida* species involved in fungal infections of the oral cavity [17–19]. Natural products include essential oils and their constituents [20, 21], which may belong to several classes of compounds, but terpenes and phenylpropenes are the most common [16].

The interest in isolated monoterpenes has grown over the past years as a result of their pharmacological use. This increase in interest is particularly true for thymol, which is a known antimicrobial agent [22, 23]. The use of individual components provides greater predictability, allowing the minimization of adverse effects [24].

Thymol (2-isopropyl-5-methylphenol) is a phytoconstituent classified as a monoterpene [25]. It is the majority phytoconstituent in the essential oil of thyme (*Thymus vulgaris*) [24] and is a major component of the essential oil of oregano (*Origanum vulgare*) [16]. Some studies have shown that thymol has antiseptic, anti-inflammatory, antioxidant and healing properties and a broad spectrum of biological activity [26–28].

Previous studies have shown that the toxicity of thymol, like that of any other substance, is directly related to the concentration applied to cell cultures [29]. The exposure of human fibroblasts to different concentrations of thymol (25 to 100 μg/mL) revealed cell viability above 96 % when at 24, 48 and 72 h [30]. The cytotoxic activity (50 % lethal concentration) of thymol in U-937 human promonocytic cells was LC_50_ ≥ 400 μg/mL [31].

In this context, the present study investigated the antifungal activity of thymol against *C. albicans* strains with respect to growth inhibition, microbial death, mode of action and synergistic effect in combination with the synthetic antifungal nystatin.

## Methods

### Research site

The microbiological tests were performed at the Laboratory of Oral Microbiology, Tropical Medicine Center (NUMETROP) - Center for Health Sciences, Federal University of Paraiba, Paraiba, Brazil.

### Microorganisms

The reference *Candida spp.* strains used in this study were Dutch cultures from the Central Bureau voor Schimmel (CBS; Fungi Culture Central Office): *C. albicans* CBS 562, *C. tropicalis* CBS 94 and *C. krusei* CBS 573.

### Test products

The phytoconstituent thymol provided by Quinarí® (Ponta Grossa, Parana, Brazil) and the synthetic antifungal nystatin (Sigma-Aldrich, St. Louis, USA) were used in the *in vitro* assays. The thymol presented as a white powder and was solubilized in 70°GL alcohol.

### Minimum inhibitory concentration (MIC)

The MIC was determined using the microdilution technique. Ninety-six-well microtiter plates with a U-shaped bottom were used (ALAMAR®, Diadema, São Paulo, Brazil). Initially, 100 μL of Sabouraud Dextrose Broth (SDB) (HIMEDIA®, Mumbai, India) was distributed in the plate holes. Then, 100 μL of thymol solution was transferred to the first well and serially diluted by transferring an aliquot of 100 μL from the most concentrated well to the next cavity. Thus, thymol concentrations from 5.000 μg/mL to 2.44 μg/mL were obtained. Finally, 100 μL of inoculum corresponding to each strain was added to each cavity [32].

The inocula were prepared in 0.9 % saline, and the turbidity of the fungal suspensions was compared with that of the solution in tube 0.5 of the McFarland nephelometric scale, which corresponds to an inoculum of approximately 10^6^ CFU/mL, and adjusted as needed. Then, the suspension was diluted in SDB to obtain an inoculum concentration of 10^3^ CFU/mL [32].

Meanwhile, the viability of yeast strains (growth control) and medium sterility were controlled. As a positive control, the synthetic antifungal nystatin was used. Through serial dilution, nystatin concentrations from 1,000 μg/mL to 0.48 μg/mL were obtained. As a negative control, 70°GL alcohol was diluted in water in the same proportions used for solubilization of thymol.

The test was performed in triplicate, and the microtiter plates were incubated at 35 °C for 24 h. A visual reading was performed to determine the MIC of thymol on yeast strains. The formation of cell clumps (“buds”) on the bottoms of the wells was considered. Thus, the lowest test product concentration that could visibly inhibit fungal growth was considered the MIC [32].

To confirm the presence of viable microorganisms, 10 μL of TTC dye (2,3,5-triphenyl tetrazolium chloride) was used. This dye reflects the activity of the dehydrogenase enzymes involved in the process of cell respiration [33].

### Minimum fungicidal concentration (MFC)

After the MIC determination, aliquots of the concentration corresponding to the MIC and the two immediately most concentrated concentrations (for the thymol-treated cultures and the positive and negative controls) were subcultured in Petri dishes containing Sabouraud Dextrose Agar (SDA; HIMEDIA®, Mumbai, India) and incubated at 35 °C for 48 h. The MFC was defined as the lowest product concentration that prevented visible growth; that is, the lowest concentration that was able to kill the fungus [32].

The MFC/MIC ratio was calculated to determine whether thymol has a fungistatic (MFC/MIC ≥ 4) or fungicidal activity (MFC/MIC <4) [34].

### Mode of action

The mode of action was tested to indicate whether the antifungal activity of thymol involves a direct interaction with the cell wall structure of *C. albicans* (via testing with sorbitol) or with the ion permeability of the membrane of this organism (via the test with ergosterol).

### Test with sorbitol

The MIC of thymol in the presence sorbitol (an osmotic protector) against *C. albicans* was determined using the microdilution technique [32] in triplicate.

Initially, 100 μL of SDB was added to each well of the microtiter plate. Subsequently, 100 μL of thymol solution was transferred to the first well and serially diluted with the removal of an aliquot of 100 μL from the most concentrated well to the subsequent cavity. Thymol solution concentrations from 5.000 μg/mL to 2.44 μg/mL were obtained. Then, 100 μL of fungal inoculum (10^3^ CFU/mL) prepared in SDB previously supplemented with sorbitol (Sigma-Aldrich, St. Louis, USA) was transferred to the wells for a final concentration of 0.8 M sorbitol in each well [35–37].

A microorganism control was created by placing 100 μg of SDB and 100 μg of the inoculum with sorbitol (0.8 M) in each cavity. Sterility control was also performed; 100 μg SDB with sorbitol (0.8 M) was placed in a plate column without fungal suspension. The plates were incubated at 35 °C and the results were read after 48 h and again after 7 days [35–37].

### Test with ergosterol

To determine whether thymol interacts with ergosterol, the MIC against *C. albicans* was determined using the microdilution technique [32] in triplicate in the presence and absence of exogenous ergosterol (Sigma-Aldrich, St. Louis, USA).

First, 100 μL of SDB was added to each well of the microtiter plate. Then, 100 μL of thymol solution was transferred to the first well and serially diluted by transferring a 100 μL aliquot from the most concentrated well to the next well. Thymol solution concentrations from 5.000 to 2.44 μg/mL were obtained. Subsequently, 100 μL of fungal inoculum (10^3^ CFU/mL) prepared in SDB previously supplemented with ergosterol in concentrations of 100, 200 and 400 μg/mL was transferred to the wells [35, 37].

Nystatin was tested as a positive control, and yeast growth and sterility were also controlled.

### Synergism test: checkerboard method

The combined effect of the two substances (nystatin and thymol) was determined using the checkerboard technique to derive the fractional inhibitory concentration (FIC) index.

Test product solutions of certain concentrations (determined by their respective MICs) were used. Initially, 100 μL of SDB was added to the wells. Then, 50 μL of each test product at various concentrations (MIC x 8, MIC x 4, MIC x 2, MIC, MIC ÷ 2, MIC ÷ 4, and MIC ÷ 8) was added horizontally (thymol) and vertically (nystatin). Finally, 10 μL of fungal inoculum of the tested strains was added (10^3^ CFU/mL). Growth and sterility controls were also performed. The results were read visually, and fungal growth was shown using TTC dye. The assay was performed in triplicate, and the microplate was incubated for 48 h at 35 °C [38–40].

The FIC index was calculated as the sum of FIC^A^ + FIC^B^, where A is thymol and B is nystatin. FIC^A^, in turn, is calculated using the MIC^A^ combined/MIC^A^ alone ratio, while FIC^B^ = the MIC^B^ combined/MIC^B^ alone ratio. This index was interpreted as follows: synergism (<0.5), additivity (0.5-1.0), indifference (>1) or antagonism (>4.0).

## Results

### MIC and MFC determination

The MIC and MFC values of thymol and nystatin against *Candida spp*. are shown in Table 1. The vehicle used in the thymol solution (70°GL alcohol), at the same concentration found in the solution of lower concentration of thymol which promoted fungal growth inhibition, was not effective, meaning that it did not interfere with the MIC values of the tested product against yeasts. The MIC/MFC ratio for thymol showed that it had a fungicidal effect against all of the tested strains.

**Table 1:** Antifungal activity of thymol against *Candida spp*

Strain	Thymol			Nystatin		
	MIC	MFC	MIC/MFC	MIC	MFC	MIC/MFC
	(μg/mL)	(μg /mL)	ratio	(μg/mL)	(μg/mL)	ratio
*Candida albicans*	39.0	39.0	1	1.9	1.9	1
CBS 562						
*Candida tropicalis*	78.0	78.0	1	1.9	1.9	1
CBS 94						
*Candida krusei*	39.0	39.0	1	1.9	1.9	1
CBS 573						

Notes: *MIC* Minimum inhibitory concentration, *MFC* Minimum fungicidal concentration

### Mode of action of thymol

**Fig. 1:**
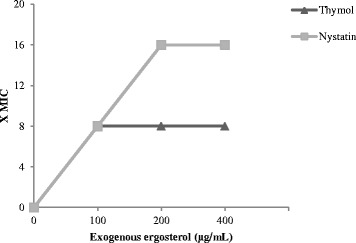
The effect of different concentrations of exogenous ergosterol (100-400 μg / mL) on the MIC (minimum inhibitory concentration) of thymol and nystatin against *Candida albicans*

The results showed that the antifungal properties of thymol are not related to the biosynthetic pathways of the cell wall because the results of the antifungal test remained unchanged in the presence of an osmotic protector (MIC in presence of sorbitol: 39.0 μg/mL). In contrast, the MIC value of thymol against *C. albicans* increased eight times (39.0 to 312.5 μg/mL) in the presence of exogenous ergosterol (Fig. 1), which indicates that thymol appears to bind to the ergosterol in the membrane, which increases ion permeability and ultimately results in cell death. The same mode of action was also observed for synthetic antifungal nystatin, which was used as a positive control.

### Synergism test

As a result of combining thymol with nystatin, a significant reduction in the MIC values was observed. For both products, the MIC reduction was 87.4 % for the three *Candida* strains tested. The FIC index value was 0.25, indicating a synergistic effect (FIC < 0.5) for this association in relation to the growth inhibition of the tested strains (Table 2).

**Table 2:** The MIC of thymol and nystatin, when used in association, and their combined FIC index values

Strain	MIC of thymol	MIC of nystatin	FIC	Interpretation
	(μg/mL)	(μg/mL)		
*Candida albicans*	4.88	0.24	0.25	Synergism
CBS 562				
*Candida tropicalis*	9.76	0.24	0.25	Synergism
CBS 94				
*Candida krusei*	4.88	0.24	0.25	Synergism
CBS 573				

Note: *MIC* Minimum inhibitory concentration, *FIC* Fractional inhibitory concentration index

## Discussion

The high prevalence and severity of infections caused by yeasts of the genus *Candida*, which can cause significant morbidity and mortality in affected patients (usually hospitalized), has encouraged investigations to elucidate new therapeutic approaches to treat candidiasis [41]. Because the oral cavity includes Candida spp. in its normal microbiota and because this disease is considered an opportunistic infection, is an important site of disease development.

This study used the Candida species usually identified in oral fungal infections. *C. albicans* is present in the oral cavities of approximately 30 to 50 % of people. It is among the most virulent species [42]; it is able to produce phospholipases and proteases that can destroy host tissues [43], and it expresses genes that are responsible for the cellular responses involved in invasive growth, cell wall formation, adaptation to osmotic stress [4] and resistance to currently available antifungal agents in the form of efflux pumps [44, 45], changes in the drugs’ site of action [46], and changes in the lipid composition of the fungal plasma membrane, which prevent the drugs’ inflow into the cell [47].

*C. tropicalis* and *C. krusei* are also important pathogens that contribute to the development of the disease. Strains that are resistant to available therapeutic agents have begun to appear, and there has been an increasing number of immunocompromised people and increasingly frequent use of antifungal agents for treatment and/or prophylaxis [48, 49].

Low thymol concentrations were needed to inhibit the growth of *Candida* strains, and thymol’s fungicidal effect is considered important for infection control because the immune responses of the host are usually compromised, making it difficult for affected patients to recover their health. The anti-*Candida* effect promoted by thymol has been reported in other studies [50, 51]. Previous studies have shown that thymol is able to significantly reduce the number of viable *C. albicans* cells, reducing up to 82 % the biofilm mass formed by this microorganism [52].

The elucidation of the action mechanisms of agents with pharmacological potential, whether of natural or synthetic origin, contributes to the development of rational therapeutic approaches, particularly in terms of infections caused by resistant microorganisms, which frequently require combinations of drugs or the use of new drugs when the first-choice agent is not effective. The results of this study suggest that thymol acts at the level of the fungal plasma membrane by interfering with the process of ergosterol biosynthesis, promoting increased membrane permeability and causing the depletion of components essential to fungal cell survival [23, 25].

Generally, terpenes, particularly monoterpenes such as thymol, are characterized by the chemical formula C_10_H_16_, which may be cyclic or branched. The chemical configuration of these molecules gives them hydrophobic properties and allows them to deposit on the lipophilic structures of microorganisms such as the plasma membrane; this deposition leads to increased permeability with a consequent loss of the electrolytes essential to cell survival [20]. Other action mechanisms may be involved, such as the inhibition of spore germination, fungal proliferation and cell respiration [53].

Natural products with intrinsic antimicrobial activity or products that promote the activity of commonly used antibiotic/antifungal agents may represent new ways to combat multiresistant microorganisms and prevent the contact of these microorganisms with synthetic products, thus reducing the risk of selecting new or improved resistance mechanisms [54]. Natural products may also be combined with traditional antimicrobials to enhance the antimicrobial activity of both [55].

According to the literature, there are scientific evidences for the use of nystatin in the treatment of fungal infections of the oral mucosa [56] and it has been indicated, with advantages over the other antifungals, such as topical use and less side effects [57]. However, fungal resistance has been reported [57] and the association between synthetic and natural antifungal is an alternative to reducing the dose required for the effect and may reduce undesired side effects and prevent the development of resistance to antifungals [58].

In the laboratory tests performed to investigate the antimicrobial activity of synthetic or natural agents, one of the simplest and best known protocols is the checkerboard test, which provides a two-dimensional arrangement of different concentrations of substances and allows the calculation of the FIC, which necessary for assessing the combination’s synergism, additivity, indifference or antagonism [59].

To promote greater efficiency of thymol and nystatin when used at lower concentrations, the association of these substances was proposed, and synergistic effect was observed, with reduction of 87.4 % of the concentrations of both products in relation to the effect provided when assessed separately.

There are several mechanisms involved in the synergistic activity of antifungal agents: a) the inhibition of different stages in the fungal intracellular pathways that are essential for cell survival; b) increased penetration of one antifungal agent resulting from the action of another antifungal agent on the fungal cell membrane; c) the inhibition of carrier proteins; and d) the simultaneous inhibition of different cell targets [60]. The mechanism of combined thymol and nystatin appears to involve the inhibition of ergosterol formation as a result of the antifungal agents’ action on the different enzymes responsible for the biosynthesis of ergosterol and/or because an increase in cell permeability allows the passage of one or both agents.

It is noteworthy that this is the first study that reports the synergistic effect of combined thymol and nystatin and its potential use to treat superficial infections in the oral mucosa caused by *Candida* species. Therefore, the findings described here encourage the development of clinical trials to evaluate the efficacy of this combination treatment.

## Conclusions

From the results obtained, it was concluded that thymol has fungicidal action against *C. albicans*, *C. tropicalis* and *C. krusei* strains, and its effect is probably the result of interference with the synthesis and/or presence of ergosterol in the plasma membrane. The combination of thymol and nystatin had a synergistic effect for inhibiting the growth of these strains.

## Abbreviations

*C. albicans*: *Candida albicans*
*C. krusei*: *Candida krusei*
*C. tropicalis*: *Candida tropicalis*
CBS: Central Bureau voor Schimmel
CFU: Colony-forming unit
FIC: Fractional inhibitory concentration index
MFC: Minimum fungicidal concentration
MIC: Minimum inhibitory concentration
NUMETROP: Tropical Medicine Center, Center for Health Sciences, Federal University of Paraiba, Paraiba, Brazil
SDA: Sabouraud Dextrose Agar
SDB: Sabouraud Dextrose Broth
TTC (dye): 2,3,5-triphenyl tetrazolium chloride
